# Transmembrane and Ubiquitin-Like Domain-Containing Protein 1 (Tmub1/HOPS) Facilitates Surface Expression of GluR2-Containing AMPA Receptors

**DOI:** 10.1371/journal.pone.0002809

**Published:** 2008-07-30

**Authors:** Hyunjeong Yang, Hiroshi Takagi, Yoshiyuki Konishi, Hiroshi Ageta, Koji Ikegami, Ikuko Yao, Showbu Sato, Ken Hatanaka, Kaoru Inokuchi, Dae-Hyun Seog, Mitsutoshi Setou

**Affiliations:** 1 Department of Biological Information, Tokyo Institute of Technology, Yokohama, Kanagawa, Japan; 2 Mitsubishi Kagaku Institute of Life Sciences, Machida, Tokyo, Japan; 3 Department of Biochemistry, College of Medicine Inje University, Busan, Korea; 4 Department of Molecular Anatomy, Hamamatsu University School of Medicine, Hamamatsu, Shizuoka, Japan; National Institutes of Health, United States of America

## Abstract

Some ubiquitin-like (UBL) domain-containing proteins are known to play roles in receptor trafficking. Alpha-amino-3-hydroxy-5-methyl-4-isoxazole propionic acid receptors (AMPARs) undergo constitutive cycling between the intracellular compartment and the cell surface in the central nervous system. However, the function of UBL domain-containing proteins in the recycling of the AMPARs to the synaptic surface has not yet been reported.

Here, we report that the Transmembrane and ubiquitin-like domain-containing 1 (Tmub1) protein, formerly known as the Hepatocyte Odd Protein Shuttling (HOPS) protein, which is abundantly expressed in the brain and which exists in a synaptosomal membrane fraction, facilitates the recycling of the AMPAR subunit GluR2 to the cell surface. Neurons transfected with Tmub1/HOPS-RNAi plasmids showed a significant reduction in the AMPAR current as compared to their control neurons. Consistently, the synaptic surface expression of GluR2, but not of GluR1, was significantly decreased in the neurons transfected with the Tmub1/HOPS-RNAi and increased in the neurons overexpressing EGFP-Tmub1/HOPS. The altered surface expression of GluR2 was speculated to be due to the altered surface-recycling of the internalized GluR2 in our recycling assay. Eventually, we found that GluR2 and glutamate receptor interacting protein (GRIP) were coimmunoprecipitated by the anti-Tmub1/HOPS antibody from the mouse brain.

Taken together, these observations show that the Tmub1/HOPS plays a role in regulating basal synaptic transmission; it contributes to maintain the synaptic surface number of the GluR2-containing AMPARs by facilitating the recycling of GluR2 to the plasma membrane.

## Introduction

Proteins can be modified by either a single ubiquitin moiety or polymeric ubiquitin chains to alter their stability, localization, binding partners, or physical conformation [1], [2]. Ubiquitination has been reported to regulate cell surface receptors [3], such as AMPARs [4], and γ-aminobutyric acid A receptors (GABA_A_Rs) [5]. Like ubiquitin, UBL proteins and UBL domain-containing proteins appear to regulate a wide variety of proteins of various processes [6], [7]. UBL proteins share the three-dimensional structure and conjugation properties of ubiquitin, while UBL domain-containing proteins are not conjugatable and are found in larger multidomain proteins [8]. Some UBL proteins and UBL domain-containing proteins have been reported to be involved in receptor regulation. One of the UBL domain-containing proteins, Plic-1/ubiquilin-1, regulates the cell surface number and subunit stability of GABA_A_Rs [9]. Moreover, the GABA_A_R-associated protein (GABARAP/ubiquilin-2), which contains a UBL core domain in the C-terminus [10], traffics GABA_A_Rs to the plasma membrane in neurons [11].

Synaptic function is regulated by various processes, including the transport of proteins [12]–[14], the release of neurotransmitters [15], post-translational modification of microtubules [16], [17], local translation of dendritic RNA [18], and the ubiquitination of proteins [19]. In the postsynaptic regions of excitatory synapses, a precise AMPAR trafficking is crucial for synaptic transmission [20]. AMPARs, which form tetramers, consist of GluR1–4 subunits [21]. In the adult hippocampus, GluR1/GluR2 and GluR2/GluR3 complexes are predominant [22]. GluR1/GluR2 travel fast only under conditions of stimulation, while GluR2/GluR3 are recycled constitutively between the intracellular compartment and the cell surface [23], [24]. Although the precise regulation of AMPAR recycling is critical for the maintenance of postsynaptic transmission, the underlying mechanisms remain elusive.

Here, we introduce a transmembrane and ubiquitin-like domain-containing protein as a factor for AMPAR recycling. The protein was screened from *in silico* research, by its neuronal expression and domain characteristics; UBL domain and transmembrane domains. We found that the protein is related to the recycling pathway of GluR2-containing AMPAR complexes and consequently contributes to the maintenance of the basal synaptic transmission of AMPARs.

## Results

### Tmub1/HOPS, a UBL domain-containing protein, is abundantly expressed in mouse brain

**Figure 1:**
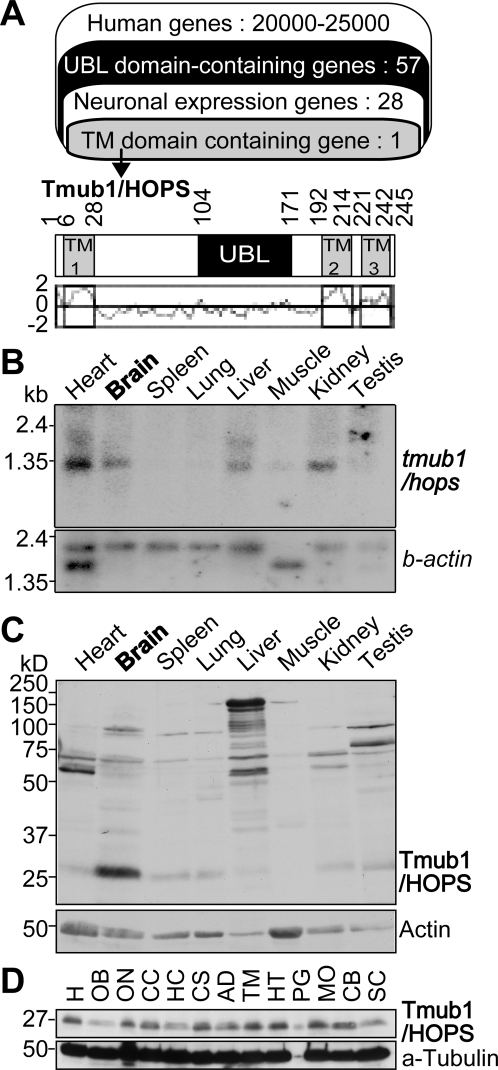
Tmub1/HOPS protein is abundantly expressed in mouse brain. (A) The summary of the *in silico* screening (upper panel) and the organization of mouse Tmub1/HOPS (lower panel). Among 20000–25000 human genes from the Celera human genome database, 57 genes contained the UBL domain (black), and 28 of them showed neuronal expression (Table S1). Among them, only one gene, Tmub1/HOPS, had putative TM domains (gray) from SOSUI. Tmub1/HOPS had 3 putative TM domains (TM1–3) and a UBL domain. The hydrophobicity profile of Tmub1/HOPS is shown (under the scheme). The X-axis corresponds to the amino acid sequences and the Y-axis, to its hydrophobicity. Abbreviations: TM, transmembrane. (B) *tmub1/hops* mRNA expression in mouse tissues. *tmub1/hops* signals were detected at approximately 1.3 kb in the heart, brain, liver, and kidney. Beta-actin was used as a loading control. (C) Western blotting of mouse tissues with anti-Tmub1/HOPS antibody. The Tmub1/HOPS protein was most abundantly expressed in the brain. The Tmub1/HOPS signals were detected at approximately 26–27 kDa. Actin was used as a loading control. (D) Western blotting of parts of the mouse brain. The Tmub1/HOPS protein was expressed in almost all parts of the mouse brain. Abbreviations: H, homogenate; OB, olfactory bulb; ON, optic nerve; CC, cerebral cortex; HC, hippocampus; CS, corpus striatum; AD, amygdala; TM, thalamus; HT, hypothalamus; PG, pituitary gland; MO, medulla oblongata; CB, cerebellum; SC, spinal cord.

In order to identify the functionally unknown UBLs in the brain, we performed bioinformatic analyses using the Celera human genome database [25] and found 57 UBLs. Among them, 28 UBLs showed neuronal tissue expression, which was confirmed by the functional annotations of mouse-3 (FANTOM3) database (Figure 1A upper panel and Table S1). Intriguingly, we found that only one of them contained putative transmembrane domains as expected by the SOSUI system, which is a tool for secondary structure prediction from protein sequences (Figure 1A lower panel). From further *in silico* search, it was predicted that the hydrophilic region is directed toward the cytoplasm and the first transmembrane domain serves as a signal peptide (data not shown). The identified protein was “Transmembrane and ubiquitin-like domain-containing protein 1 (Tmub1),” which is an official name of the gene, mRNA and protein of NCBI. This protein is also known as “Hepatocyte Odd Protein Shuttling (HOPS)” in NCBI and was previously reported as the protein related with cellular proliferation in the liver [26], [27]. We refer to this protein as Tmub1/HOPS because it represents well the domain characteristics which used in our identification. Since the Tmub1/HOPS amino acid sequences were highly conserved (89% identity) between the human and mouse genomes, we used mouse brain cDNA libraries for cloning the full-length gene.

First, in order to confirm that *tmub1/hops* mRNA is expressed in the brain, we performed northern blot analysis using RNA blots from mouse tissues. We detected clear signals at approximately 1.1 kb, a size corresponding to the *tmub1/hops* mRNA length reported in the FANTOM3 database, from the brain as well as other tissues including the heart, liver, and kidney (Figure 1B). Next, in order to examine whether the Tmub1/HOPS protein is expressed in the brain, we developed a rabbit polyclonal antibody against 29–191 amino acids of the Tmub1/HOPS protein fused with a glutathione S-transferase (GST) tag at its N terminus. By using this antibody, we performed western blotting analysis on various mouse tissues. Major signals were detected at approximately 26–27 kDa, the corresponding size expected from *tmub1/hops* mRNA. Although a strong high-molecular band was observed in the liver, there were no such signals in the brain tissue. It is possible that there is a liver-specific high-molecular factor with a similar amino sequence or conformation that is recognized easily by anti-Tmub1/HOPS antibodies. While almost all the tissues examined exhibited Tmub1/HOPS signals, the brain tissue showed the strongest signal among all (Figure 1C). Moreover, in the brain tissue, the Tmub1/HOPS protein was widely expressed (Figure 1D).

### Tmub1/HOPS is found in synaptosomal membrane

**Figure 2:**
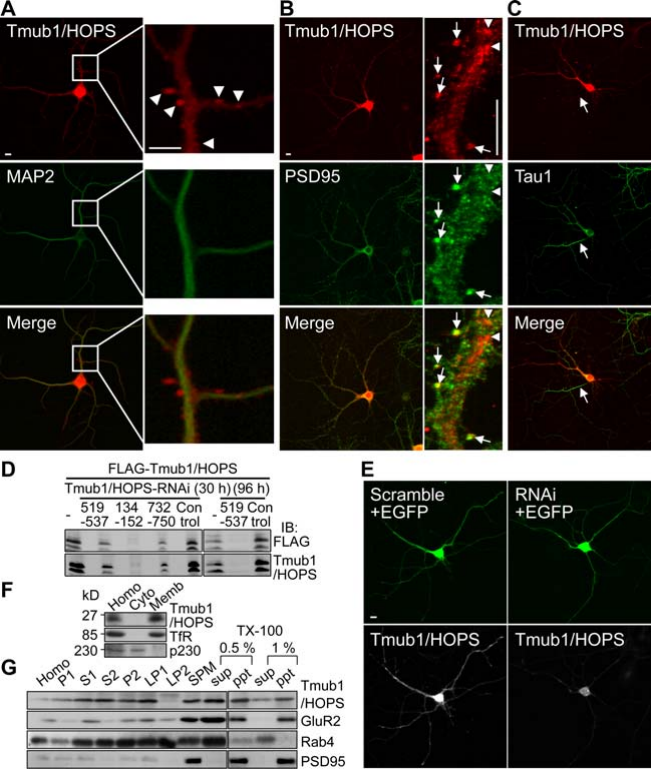
Tmub1/HOPS is observed in the synaptic membranous compartment. (A) Double immunostaining of Tmub1/HOPS along with the dendritic marker MAP2 using cultured rat hippocampal neurons. Endogenous Tmub1/HOPS signals were detected widely throughout the neuron including the soma and dendrites. In the magnified image, Tmub1/HOPS signals are found at dendritic shaft as well as at protrusion structures of dendrites (arrow heads). (B) Double immunostaining of Tmub1/HOPS along with the postsynaptic marker PSD-95. In the magnified image, some portion of Tmub1/HOPS were observed on PSD-95-positive puncta (arrows), while another portion of Tmub1/HOPS were observed in dendritic shaft as well (arrow heads). (C) Double immunostaining of Tmub1/HOPS along with the axonal marker Tau1. Tmub1/HOPS signals were hardly found in Tau1-positive axon (arrow). (D) Western blotting of HEK293 cells transfected with Tmub1/HOPS-RNAi plasmids (519–537, 134–152, 732–750) and the scramble plasmids (control). Overexpressed FLAG-Tmub1/HOPS appeared as double bands. The bands almost completely disappeared in cells transfected with Tmub1/HOPS-RNAi (134–152) for 30 h, while the control cells showed a strong expression of FLAG-Tmub1/HOPS. The cells transfected with Tmub1/HOPS-RNAi (519–537) also showed significant reduction of FLAG-Tmub1/HOPS after 96 h. (E) Neurons transfected with Tmub1/HOPS-RNAi (134–152) or its scrambled plasmid together with the EGFP plasmid for visualization (green). In the RNAi-induced neurons, Tmub1/HOPS signals (white) were strongly reduced, although faint signals still remained in the cell body; in contrast, scrambled plasmid-transfected neurons exhibited clear Tmub1/HOPS signals in the cell body and neurites. Scale bar, 10 µm. (F) Mouse brain separation into the cytosolic and membranous fractions. Most of the Tmub1/HOPS was separated into the membranous fraction. The transferrin receptor (TfR) and p230 were used as markers for the membranous and cytosolic fractions, respectively. Abbreviations: Homo, homogenate; Cyto, cytosolic; Memb, membranous. (G) Subcellular fractionation using mouse brains. The Tmub1/HOPS signals were found in the synaptosomal membrane fraction (SPM). From the SPM fraction, some of Tmub1/HOPS, GluR2, and an endosomal marker Rab4 were dissolved by TX-100, while PSD-95 was not. Abbreviations: Homo, homogenate; P1, crude nuclear; S1, crude synaptosomal; S2, cytosolic synaptosomal; P2, crude synaptosomal pellet; LP1, crude synaptosomal membrane; LP2, synaptosomal vesicle; SPM, synaptosomal membrane; S, supernatant; P, pellet.

Next, we examined whether the Tmub1/HOPS protein is expressed in neurons. We immunostained primary hippocampal cultured neurons on day *in vitro* (DIV) 14 with the anti-Tmub1/HOPS antibody. Clear Tmub1/HOPS signals were detected in the MAP2-positive dendrites and in the cell bodies of the hippocampal neurons (Figure 2A left). The magnified images of the dendrites (Figure 2A right) show that the signals are observed close to or attached to the dendrites and at the dendritic shaft (arrowheads). The signals of Tmub1/HOPS were found in PSD95-positive postsynaptic spines (Figure 2B arrows) as well as in the dendritic shaft (Figure 2B arrow heads). Further, the Tmub1/HOPS signals were hardly found in the Tau1-positive axon (Figure 2C arrow). In order to confirm that these signals show endogenous Tmub1/HOPS, we constructed Tmub1/HOPS-RNAi plasmids having sequences corresponding to 519–537 base pair (bp), 134–152 bp, and 732–750 bp of Tmub1/HOPS. In human embryonic kidney (HEK) 293 cells, overexpressed FLAG-Tmub1/HOPS was detected as two bands by anti-FLAG antibody as well as by the anti-Tmub1/HOPS antibody (Figure 2D), indicating that Tmub1/HOPS is cleaved at the C-terminus, as has been previously reported [26]. The FLAG-Tmub1/HOPS signals were reduced in cells transfected with the plasmid for Tmun1/HOPS RNAi, while the signals were normally presented in cells transfected with the scrambled plasmid. The plasmids containing 134–152 bp of Tmub1/HOPS showed the most significant RNAi effect. The plasmids containing 519–537 bp of Tmub1/HOPS also showed a significant reduction after 96 h (Figure 2D). When we introduced the RNAi of Tmub1/HOPS (134–152 bp) to the hippocampal neurons at DIV 14 and incubated them for additional 2 d, the signals detected by the anti-Tmub1/HOPS antibody were significantly reduced throughout the entire neurons, although a few signals of Tmub1/HOPS were still observed in the cell body (Figure 2E). Thus, the Tmub1/HOPS signals in the neurons shown in the immunostaining that used the anti-Tmub1/HOPS antibody were proved to be the endogenous signals of Tmub1/HOPS.

Tmub1/HOPS was expected to possess transmembrane domains exhibiting a high hydropathy value (Figure 1A lower). To determine whether the endogenous Tmub1/HOPS is localized in the membranous compartment, we fractionated mouse brain extracts into cytosolic and membranous fractions (Figure 2F). Tmub1/HOPS was fractionated into the membranous fraction rather than into the cytosolic fraction.

Because some of the Tmub1/HOPS signals were observed at the PSD95-positive postsynaptic spines (Figure 2B) and Tmub1/HOPS was localized in the crude membranous fraction (Figure 2F), we speculated that Tmub1/HOPS may exist in synaptic membrane compartments. To check this, we performed subcellular fractionation of mouse brain (Figure 2G). A crude synaptosomal pellet (P2), containing Tmub1/HOPS, was divided into a crude synaptosomal membrane (LP1) and a synaptosomal vesicle (LP2). The LP1, where much of Tmub1/HOPS was contained, was further purified into a synaptosomal membrane (SPM). Tmub1/HOPS was found in the SPM, showing that Tmub1/HOPS exists in synaptosomal membrane compartments. Then, the SPM fraction, wherein Tmub1/HOPS as well as GluR2, Rab4, and postsynaptic density protein (PSD)-95 were contained, was attempted to be dissolved by Triton X (TX)-100. Much of Tmub1/HOPS, GluR2, and Rab4 were dissolved by TX-100, while PSD-95 was not. Taken together, these results indicated that some of Tmub1/HOPS exist in the post synaptic membranes containing not the stable components of the PSD but the TX-100-soluble components, such as endosomal membranes.

### Tmub1/HOPS-RNAi decreases the amplitude of AMPAR-mediated synaptic current and increase the inward rectification of EPSC current/voltage

**Figure 3:**
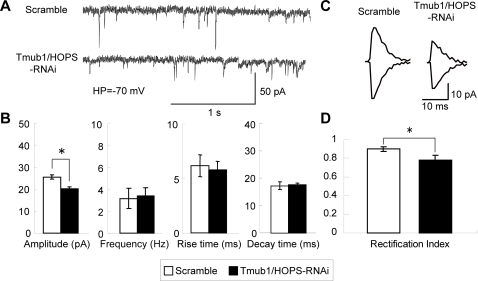
The amplitude of AMPA-mEPSC is suppressed in Tmub1/HOPS-RNAi neurons in a GluR2-containing AMPAR dependent manner. (A) Representative traces of AMPAR-mediated mEPSC. (B) The AMPA mEPSC amplitude of the Tmub1/HOPS-RNAi neuron was significantly smaller than that of the scramble-treated neuron (n = 10; *P<0.05; *t*-test). In contrast, the AMPA mEPSC frequency, rise time, and decay time of the Tmub1/HOPS-RNAi neuron did not differ significantly from those of the scramble-treated neuron (n = 10; *P*>0.05; *t*-test). (C) Averaged mEPSC at holding potential of +50 (top) and −70 mV (bottom) recorded from the control and HOPS-RNAi induced primary cultured neuron. (D) Rectification index of the HOPS-RNAi induced neuron showed the significant decrease compared to the control neuron (n = 10; *P>0.05; *t*-test).

Next, we examined whether Tmub1/HOPS plays a role in synaptic function. In the central nervous system, a majority of rapid excitatory synaptic transmission is mediated by AMPAR [21]. Therefore, we attempted to measure AMPAR-mediated synaptic transmission in the neurons transfected with the Tmub1/HOPS-RNAi and in their control neurons. Transfection was performed on DIV 14 and a miniature excitatory postsynaptic current (mEPSC) was recorded under a whole-cell voltage-clamp condition (holding potential, −70 mV and +50 mV) in the presence of 0.5 µM TTX after 2 d [15], [19]. Membrane resistance and capacitance did not differ significantly (n = 10; *P*>0.05; *t*-test) among or within the cells that were compared. This implies that Tmub1/HOPS expression itself does not alter the membranous characteristics of neurons. Figure 3A shows the representative traces of mEPSC recorded from the scramble-transfected neurons and that from the Tmub1/HOPS-RNAi-transfected neurons. The mEPSC amplitude was significantly reduced in the Tmub1/HOPS-RNAi-transfected neurons as compared to that in the scramble-transfected neurons (n = 10; *P*<0.05; *t*-test; Figure 3B). No significant differences were found in the frequency, rise time, and decay time of mEPSC. This result showed that Tmub1/HOPS played a role in maintaining AMPAR-mediated basal synaptic transmission.

To determine whether changes occur in the GluR2-containing AMPARs, we measured the AMPA-mEPSC under the voltage clamp condition in Tmub1/HOPS-RNAi neurons and the corresponding control neurons. It has been known that GluR2-lacking AMPARs show inward rectification of EPSC current/voltage relationships, while GluR2-containing AMPARs do not show the inward rectification [28]. The AMPAR-mediated mEPSCs were recorded at different holding potentials of −70 mV and +50 mV. The Tmub1/HOPS-RNAi-induced neurons (Rectification Index: 0.89±0.02; n = 10) exhibited significantly larger inward rectification than the scramble-induced neurons (Rectification index: 0.78±0.05; n = 10) (Figure 3C, D). This result indicated that Tmub1/HOPS-RNAi affects GluR2-containing AMPARs specifically rather than GluR2-lacking AMPARs.

### Tmub1/HOPS regulates synaptic surface expression of GluR2 but not GluR1

**Figure 4:**
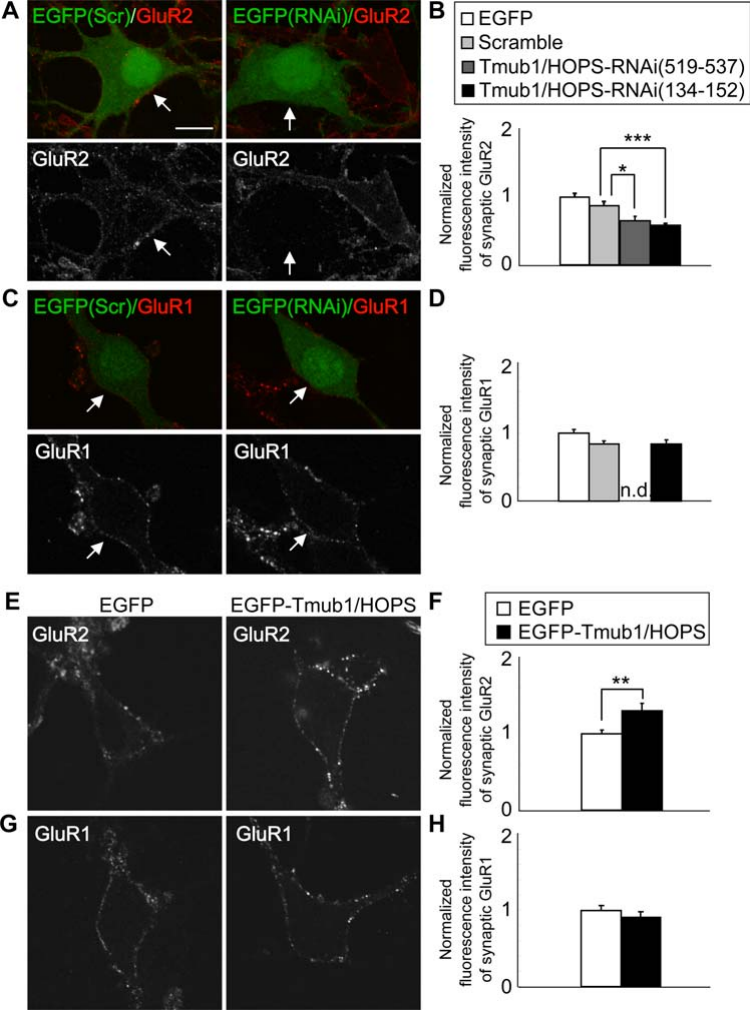
Tmub1/HOPS facilitates the surface expression of GluR2, but not of GluR1. Surface GluR2 or GluR1 of live neurons was stained with the antibody that recognizes the extracellular domain of GluR2 or GluR1. (A–D) Neurons induced by either Tmub1/HOPS-RNAi (134–152) or scramble plasmid. pSUPER-RNAi plasmids were cotransfected on DIV 14 of the cultured rat hippocampal neurons with EGFP plasmids for visualization and were incubated for follwing 2 days. (A) Cell surface GluR2 was decreased in the Tmub1/HOPS-RNAi-transfected neurons as compared with the scramble-transfected neurons. (B) The postsynaptic surface GluR2 was significantly decreased in the Tmub1/HOPS-RNAi neurons (RNAi (519–537), n = 25, *P<0.05; RNAi (134–152), n = 160, ***P<0.001, *t*-test). For postsynaptic measurement, presynaptic staining of VAMP2 or synaptophysin was performed after permeabilization, and the synaptic AMPAR fluorescence intensity was measured by measuring colocalizing presynaptic marker-positive puncta. (C, D) GluR1 staining at the cell surface did not differ between the Tmub1/HOPS-RNAi neurons and the scramble neurons. Consistent results were obtained from postsynaptic GluR1 (n = 170; *P*>0.05; *t*-test). Abbreviations: n.d., no data. (E–H) Neurons expressing either EGFP-Tmub1/HOPS or EGFP. (E) Cell surface GluR2 was increased in EGFP-Tmub1/HOPS-overexpressing neurons as compared to the EGFP-overexpressing neurons. (F) Postsynaptic surface GluR2 showed a significant increase in the EGFP-Tmub1/HOPS-overexpressing neurons as compared to the EGFP-overexpressing neurons (n = 89; **P<0.01; *t*-test). (G) In the cell surface GluR1, no significant changes were observed between EGFP-Tmub1/HOPS- and EGFP-overexpressing neurons. (H) Postsynaptic surface GluR1 did not show significant changes in EGFP-Tmub1/HOPS-overexpressing neurons as compared to EGFP-overexpressing neurons (n = 71; *P*>0.05; *t*-test). The immunofluorescence level of synaptic AMPARs was normalized by the fluorescence intensity of synaptic AMPARs on the neurons expressing EGFP. The values shown indicate the means±SEM. Scale bar, 10 µm.

The decrease in the amplitude of AMPAR mEPSC in the Tmub1/HOPS-RNAi neurons suggested a reduction in the AMPAR number on the postsynaptic surface of the Tmub1/HOPS-RNAi neurons. To examine whether the AMPAR number on the postsynaptic surface was actually decreased in the Tmub1/HOPS-RNAi neurons, we measured the immunofluorescent intensity of surface GluR2 or GluR1. In the Tmub1/HOPS-RNAi neurons, the GluR2 level was significantly decreased at the cell surface (Figure 4A) while the GluR1 level remained unchanged, as compared to their control neurons (Figures 4C). Similar results were obtained from the postsynaptic AMPARs by measuring the postsynaptic puncta, which were defined as presynaptic marker (VAMP2 or synaptophysin)-positive puncta. The fluorescence level of postsynaptic GluR2 was significantly decreased in the Tmub1/HOPS-RNAi neurons as compared to the control neurons (RNAi (519–537), n = 25, *P*<0.05; RNAi (134–152), n = 160, *P*<0.001; *t*-test; Figure 4B). On the other hand, the surface GluR1 presented no significant changes (RNAi (134–152), n = 170, *P*>0.05; *t*-test; Figure 4D). Immunostaining of synaptic AMPARs in the Tmub1/HOPS-RNAi and control neurons supported the electrophysiological results that the decrease in the amplitude is due to the reduction in the AMPAR number, particularly the GluR2 number.

To confirm the Tmub1/HOPS-dependent changes of the AMPAR surface expression, we evaluated the surface expression of the AMPARs on the Tmub1/HOPS-overexpressing neurons (Figures 4E, G). The EGFP-Tmub1/HOPS-overexpressing neurons showed a significant increase in surface GluR2 as compared with that on the EGFP-overexpressing neurons (n = 89, *P*<0.01, *t*-test; Figure 4F); in contrast, the EGFP-Tmub1/HOPS-overexpressing neurons did not show any significant differences in the surface GluR1 level (n = 71, *P*>0.05; *t*-test; Figure 4H). These observations suggested that Tmub1/HOPS regulates the surface expression of GluR2 but not of GluR1.

### Recycling of internalized GluR2 to cell surface is delayed in Tmub1/HOPS-RNAi neurons

AMPARs have been known to recycle constitutively between the plasma membrane and the intracellular compartment [23], [24]. Since Tmub1/HOPS expression altered the synaptic surface expression of GluR2, we hypothesized that Tmub1/HOPS played a role in the recycling pathway of GluR2-containing AMPARs; hence, we performed a recycling assay for GluR2 and GluR1 in the Tmub1/HOPS-RNAi neurons and their control neurons.

**Figure 5:**
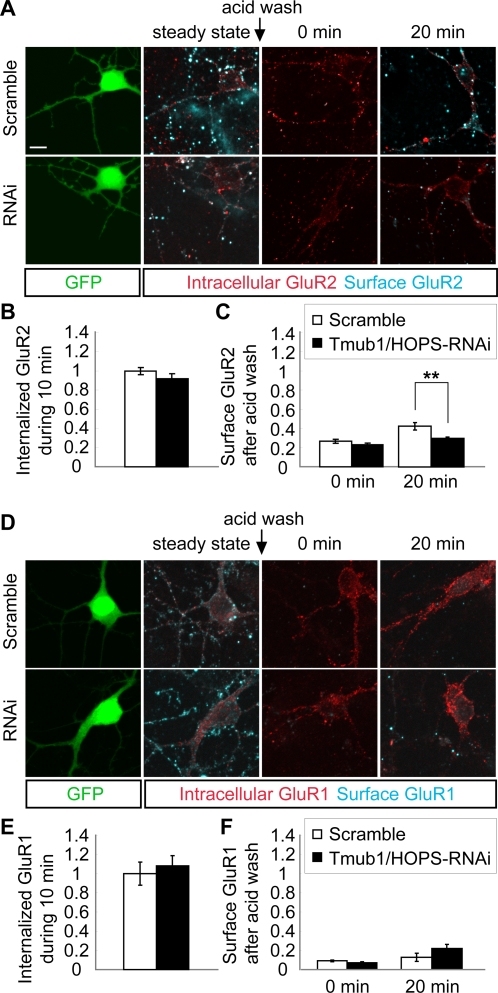
Tmub1/HOPS-RNAi decreases the recycling of internalized GluR2, but not of GluR1, to the cell surface. (A) The representative images of the GluR2 recycling assay on Tmub1/HOPS-RNAi and scramble-transfected neurons. Live neurons were stained with the anti-GluR2 antibody and were incubated for 10 min for internalization. After the internalization period, the antibodies remaining on the surface were removed using an acid buffer. Then, the neurons were further incubated for returning of the antibody-GluR2 complex to the cell surface. After fixation, the surface-recycled GluR2 was detected with a secondary antibody, and the neurons were then permeabilized followed by the detection of intracellular GluR2 with another secondary antibody. (B) The normalized value of internalized GluR2 during the first 10 min. The internalized GluR2 level did not differ significantly between the Tmub1/HOPS-RNAi- and scramble-transfected neurons (n = 12; *P*>0.05; *t*-test). (C) The normalized value of surface GluR2 depending on duration of incubation after the acid wash. After the incubation of 20 min, the recycling of internalized GluR2 to the cell surface was significantly delayed in Tmub1/HOPS-RNAi-transfected neurons as compared to the scramble-transfected neurons (n = 27; **P<0.01; *t*-test). (D) The representative images of the GluR1 recycling assay on the Tmub1/HOPS-RNAi- and scramble-transfected neurons. (E) The normalized value of internalized GluR1 during the first 10 min. The internalized GluR1 level did not differ significantly between the Tmub1/HOPS-RNAi- and scramble-transfected neurons (n = 9; *P*>0.05; *t*-test). (F) The normalized value of surface GluR1 depending on the duration of incubation after the acid wash. After the incubation of 20 min, the recycling of internalized GluR1 did not differ significantly between the Tmub1/HOPS-RNAi-transfected and scramble-transfected neurons (n = 9; *P*>0.05; *t*-test). The fluorescence intensity was normalized by the intensity of the internalized AMPARs during the first 10 min in the scramble-transfected neurons. The values shown indicate the means±SEM. Scale bar, 10 µm.

After labeling of surface GluR2 or GluR1, the cells were incubated for 10 min to allow the internalization of the receptor-antibody complexes. After the internalization period, the cells for “steady state” were fixed. The remaining surface antibodies were stripped away using an acid buffer. After the acid wash, the cells for “0 min” were fixed. The other cells were further incubated at 37°C to allow the recycling of the internalized receptor-antibody complex to the cell surface. After 20 min of further incubation, the cells for “20 min” were fixed. The fixed cells were stained with a secondary antibody for labeling the surface receptor under impermeable conditions, and then, the cells were permeabilized for labeling of the intracellular receptors with another secondary antibody under permeable conditions (Figures 5A, D). The fluorescence intensity from the cell body and the dendrites was measured, and similar results were obtained from both the regions. The graphs shown in Figure 5 represent the results of the measurement in dendrites.

During the first 10 min of the internalization period, there were no significant differences in the internalized GluR2 between the Tmub1/HOPS-RNAi-transfected and scramble-transfected neurons (n = 12, *P*>0.05; *t*-test; Figure 5B). In contrast, the level of surface-recycled GluR2 in the Tmub1/HOPS-RNAi-transfected neurons was significantly delayed at 20 min after the acid wash, as compared to the scramble-transfected neurons. After incubation for 20 min, 42.3% of the internalized GluR2 was surface-recycled in the scramble-transfected neurons, while only 29.7% of it was surface-recycled in the Tmub1/HOPS-RNAi-transfected neurons (n = 27, *P*<0.01; *t*-test; Figure 5C). The same experiments were performed using the anti-GluR1 antibody (Figures 4D–F). For the first 10 min of the internalization period, the internalized GluR1 level did not differ significantly between the Tmub1/HOPS-RNAi-transfected and the scramble-transfected neurons (n = 9, *P*>0.05; *t*-test; Figure 5E). Unlike in the case of GluR2, the level of surface-recycled GluR1 during 20 min after the acid wash did not differ significantly between the Tmub1/HOPS-RNAi-transfected and the scramble-transfected neurons (n = 9, *P*>0.05; *t*-test; Figure 5F). These recycling assays of GluR2 and GluR1 revealed that Tmub1/HOPS is related to the recycling of GluR2, but not to the recycling of GluR1, to the cell surface, indicating that the reduction of the surface expression of GluR2 is due to the delayed recycling of GluR2 in neurons transfected with Tmub-RNAi.

### Recycling of internalized GluR2 to cell surface is enhanced in neurons overexpressing Tmub1/HOPS

**Figure 6:**
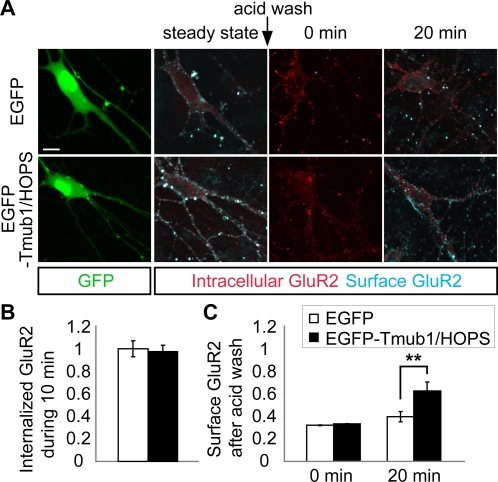
Tmub1/HOPS overexpression increases the recycling of internalized GluR2 to the cell surface. The same experiments as described in Figure 5 were performed on neurons expressing EGFP-Tmub1/HOPS or EGFP. (A) The representative images of the GluR2 recycling assay on neurons overexpressing EGFP-Tmub1/HOPS or EGFP. (B) The normalized value of internalized GluR2 during the first 10 min. The internalized GluR2 level did not differ significantly between EGFP-Tmub1/HOPS and EGFP-overexpressing neurons (n = 57; *P*>0.05; *t*-test). (C) The normalized value of surface GluR2 depending on the duration of incubation after the acid wash. After 20 min of the incubation, the level of surface-recycled GluR2 was significantly increased in the EGFP-Tmub1/HOPS-overexpressing neurons as compared to the EGFP-overexpressing neurons (n = 29; **P<0.01; *t*-test). The fluorescence intensity was normalized by the intensity of the internalized GluR2 during the first 10 min in the EGFP-overexpressing neurons. The values shown indicate the means±SEM. Scale bar, 10 µm.

We performed the same experiments under Tmub1/HOPS-overexpression conditions in order to confirm the effect of Tmub1/HOPS on GluR2 recycling (Figure 6A). The amount of internalized GluR2 during the first 10 min did not differ significantly between the neurons overexpressing EGFP and the neurons overexpressing EGFP-Tmub1/HOPS (n = 57, *P*>0.05; *t*-test; Figure 6B). In contrast, the surface-recycled GluR2 level at 20 min after the acid wash was significantly increased in the neurons overexpressing EGFP-Tmub1/HOPS as compared to the neurons overexpressing EGFP (n = 29, *P*<0.01; *t*-test; Figure 6C). This result showed that Tmub1/HOPS facilitated the recycling of the internalized GluR2 to the cell surface, verifying the effect of Tmub1/HOPS on GluR2 recycling.

### A large portion of Tmub1/HOPS colocalizes with GluR2 at recycling endosomes

In the recycling pathway of GluR2-containing AMPARs, which endosomes does Tmub1/HOPS exist at? In the recycling of AMPARs, NMDA/TTX stimulation with TTX preincubation or AMPA stimulation induces AMPARs to sort to the recycling pathway and not to degradation pathway [29], [30]. At 10 min after the stimulation, AMPARs are mainly colocalized with early endosomal markers, while at 30 min they show low colocalization with early endosomal markers but show unchangeable or further increased colocalization with recycling endosomal markers [29]–[31]. This spatiotemporal information about endosomal localization of AMPARs depending on time is proved by biochemical/immunocytochemical methods [29] and generally used in other studies [30], [31].

**Figure 7:**
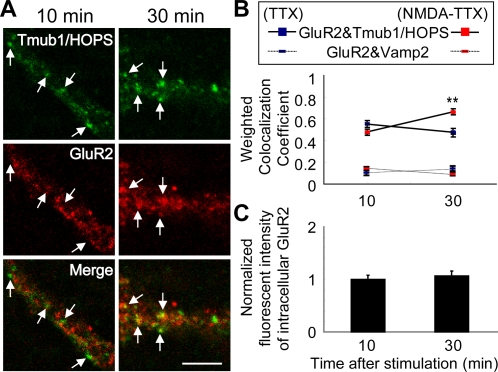
A large portion of Tmub1/HOPS colocalizes with GluR2 at recycling endosomes. After 1 h of preincubation with TTX, neurons were incubated with extracellularly binding anti-GluR2 antibody, stimulated with NMDA/TTX for 3 min, and further incubated for 10 or 30 min. Then, the cells were acid washed to remove extracellular labeling, fixed, and colabeled with Tmub1/HOPS or the synaptic vesicle protein VAMP2. (A) Tmub1/HOPS (green) and GluR2 (red) and their merged images (yellow) are shown. Many of Tmub1/HOPS-positive dots (arrows in the left image) were not colocalized with GluR2 at 10 min after NMDA stimulation, while many of the them (arrows in the right image) were colocalized with GluR2 at 30 min after the stimulation. Scale bar, 10 µm. (B) The weighted colocalization coefficient between GluR2 and Tmub1/HOPS (square) or VAMP2 (rectangle) is shown. Blue color presents control cells for TTX incubation, while red color presents cells for NMDA/TTX incubation. The weighted colocalization coefficient between GluR2 and Tmub1/HOPS in NMDA/TTX incubation was significantly increased at 30 min compared with at 10 min (n = 14 ; **P<0.01; *t*-test), while its coefficient in TTX incubation or the coefficient between GluR2 and VAMP2 in TTX or NMDA/TTX incubation did not show significant changes. (C) Normalized fluorescent intensities of intracellular GluR2 at incubation time of 10 min or 30 min are shown. The fluorescent intensity of intracellular GluR2 at 10 min was used for normalization. There was no significant change between the intensity of 10 min and the intensity of 30 min. The values shown indicate the means±SEM.

To determine in which endosomes Tmub1/HOPS is found, we used above spatiotemporal information. After the NMDA/TTX stimulation, the colocalizing ratio between Tmub1/HOPS (or VAMP2) and GluR2 was measured depending on time. Little of the Tmub1/HOPS-positive dots were colocalized with GluR2 at 10 min after the stimulation (Figure 7A arrows in the left image, 7B), while a significantly large portion of the Tmub1/HOPS-positive dots were colocalized with GluR2 at 30 min (Figure 7A arrows in the right image, 7B). The colocalizing ratio between VAMP2 and GluR2 was not significantly changed during the indicated incubating period after the stimulation. TTX incubation alone did not show any significant changes during the incubation time in the colocalizing ratio between GluR2 and Tmub1/HOPS (or VAMP2) (Figure 7B). The fluorescent intensities of intracellular GluR2 did not significantly change during the incubation time (Figure 7C). These results showed that a large portion of Tmub1/HOPS colocalizes with GluR2 at recycling endosomes during the recycling period of GluR2-containing AMPARs.

### Tmub1/HOPS is associated with GluR2 and GRIP

Our present study has demonstrated that Tmub1/HOPS regulates the recycling of GluR2-containing AMPARs. Hence, we speculated whether endogenous Tmub1/HOPS had any association with GluR2 or GluR2-interacting factors that regulate GluR2 recycling. To determine this, we subjected whole mouse brain to immunoprecipitation with the anti-Tmub1/HOPS antibody. We found that GluR2 and GRIP, a PDZ protein that interacts with GluR2 [32], were coimmunoprecipitated by the anti-Tmub1/HOPS antibody. Syntaxin 13, which regulates transferrin receptor recycling [33], was not coimmunoprecipitated by the anti-Tmub1/HOPS antibody. We also examined GluR1 but could not obtain a clear answer because of the high background noise level. This result showed that Tmub1/HOPS is associated with the complexes containing GluR2 and GRIP.

Because Tmub1/HOPS does not contain PDZ domains, we hypothesized that Tmub1/HOPS interacts with GRIP for GluR2 trafficking. While we detected the interaction between Tmub1/HOPS and GRIP in HEK293 cells, they did not bind directly in yeast 2-hybrid assay (data not shown). Although we did not obtain consistent results between the above two assays, our results showed that the endogenous Tmub1/HOPS is associated with the complexes that contain GluR2 and GRIP in the mouse brain.

## Discussion

In this study, we introduced the neuronal function of Tmub1/HOPS that we screened by *in silico* analysis. This protein was initially identified as an overexpressed protein during liver regeneration after partial hepatectomy [26]. Its overexpression interferes with protein synthesis and suppresses proliferation [26], while its depletion generates supernumerary centrosomes, multinucleated cells, and multipolar spindle formation in NIH3T3 cells [27]. This protein is found in cytosolic complexes containing gamma-tubulin and CRM-1 in hepatoma cells and has been implicated as an essential constituent of centrosome assembly [27]. The following findings of our present report are consistent with the findings of previous reports, i.e., the protein expression level of Tmub1/HOPS is low during normal conditions in the liver (Figure 1C) and that its signals show two bands on western blotting (Figure 2D). In contrast, its localization and function appear to be slightly different. Previous reports show that Tmub1/HOPS is localized at the centrosome and is important for the normal proliferation of hepatoma cells, while our present study presents that Tmub1/HOPS exists widely including in cell body/neurites and plays a role in receptor trafficking within the neuron. Gamma-tubulin, which is localized to the centrosome in cycling cells, is present at the centrosome of neurons just beginning to extend their processes, while it is not associated with centrosomes in neurons in which functional synaptic connections have formed [34]. This suggests that centrosomes exist in different fashions depending upon whether the cell is of the mitotic or postmitotic type. We investigated the localization and function of Tmub1/HOPS in neurons having functional synaptic connections. The differences in our findings and those of previous reports appear to be due to the different cell types.

From the FANTOM3 database, which provides data regarding expressed sequence tags obtained from murine tissues [35], *tmub1/hops* RNA was expressed in the brain (Table S1); this was confirmed by our northern blot analysis (Figure 1B). We confirmed that Tmub1/HOPS protein is abundantly expressed in the brain (Figure 1C), consistent with the database expectation. The expression patterns of *tmub1/hops* mRNA and the Tmub1/HOPS protein were not completely consistent with each other, suggesting that the effects of transcription, translation, and posttranslational degradation might differ among the tissues. In the brain tissue, the Tmub1/HOPS protein was widely expressed (Figure 1D), suggesting that Tmub1/HOPS may play its roles in various parts of the brain. Consistently with the *in silico* expectation that Tmub1/HOPS possesses transmembrane domains (Figure 1A), endogenous Tmub1/HOPS was found in the membranous fraction of the mouse brain extracts (Figure 2F). A previous report [36], which showed growth suppression of *Escherichia coli* by the expression of Tmub1/HOPS because of its putative transmembrane regions, also supports the fact that Tmub1/HOPS possesses transmembrane domains.

In neurons, Tmub1/HOPS, whose signals were confirmed to be endogenous ones (Figure 2D, E), was distributed in the dendrites (Figure 2A) rather than axons (Figure 2C). Further, a portion of Tmub1/HOPS was found in the post synaptic spines (Figure 2B). Our data suggested that in the post synapse, Tmub1/HOPS exists to the synaptic membranous fraction including not stable post synaptic density components but endosomal membranous components (Figure 2G). Because GluR2 is recycled between plasma membrane and intracellular compartments, some portion of GluR2 is likely to localize at endosomes, where Tmub1/HOPS is suggested to play its role in GluR2 recycling. Further, Tmub1/HOPS was revealed to exist in recycling endosomes rather than early endosomes (Figure 7). Although the Tmub1/HOPS signals in a neuron are widely distributed, our data sufficiently explain the functional existence of Tmub1/HOPS for its GluR2 regulation.

In the Tmub1/HOPS-RNAi-transfected neurons, only the amplitude, but not the frequency, rise time, and decay time, of AMPAR mEPSC was significantly decreased, (Figures 3A, B), suggesting that only the number of postsynaptic AMPARs was decreased, while the biophysical features of AMPARs remained unchanged. Furthermore, Tmub1/HOPS-RNAi-transfected neurons showed the larger inward rectification of AMPAR current than the control neuron. Our results indicated that the GluR2-containing AMPARs were mainly contributed to the modulation of AMPAR mediated basal synaptic transmission by Tmub1/HOPS (Figures 3C, D). It has been reported that peptides inhibiting the interaction between NSF and GluR2 evoked a run-down of the basal synaptic transmission, while the inhibition between AP2 and GluR2 did not affect the basal synaptic transmission [37]. Considering the previous report, Tmub1/HOPS is presumed to play a crucial role in GluR2 recycling, which is regulated by NSF but not by AP2 for the regulation of the postsynaptic GluR2-containing AMPARs. Although the amplitude change in the Tmub1/HOPS-RNAi neurons was significant as compared to that in the control neurons (Figure 3B), the extent of changes in electrophysiology was rather less than the extent of changes in the immunostaining of the postsynaptic surface GluR2 (Figure 4B), because not all AMPARs contain GluR2 [38], i.e., GluR1 homomers exist.

An approximately 35% decrease in the surface endogenous GluR2 was observed in the Tmub1/HOPS-RNAi neurons, where the interaction between Tmub1/HOPS and the AMPAR complexes was inferred to be inhibited, as compared to the control neurons (Figures 4A, B). A similar or larger reduction in surface endogenous GluR2 expression was observed in neurons that inhibit the NEEP21-GRIP [31], PICK1-GRIP [39], or NSF-GluR2 [40] interactions. No significant changes were observed in the surface endogenous GluR1 in the Tmub1/HOPS-RNAi neurons, as compared to the control neurons (Figures 4C, D), suggesting that Tmub1/HOPS selectively regulates GluR2 and not GluR1. Similarly, interference with the NEEP21-GRIP [31] or PICK1-GRIP [39] interactions did not impair the surface expression of endogenous GluR1. Our results from the Tmub1/HOPS overexpression experiments were consistent with those of the Tmub1/HOPS-RNAi experiments. The surface expression of GluR2 (Figures 4E, F), but not of GluR1 (Figures 4G, H), was increased in the Tmub1/HOPS-overexpressing neurons. Similarly, the expression of full-length GRIP enhanced the surface expression of coexpressed GluR2, suggesting that GRIP actively promotes GluR2 surface trafficking [41]. Taken together, our immunostaining results and previous reports of the AMPAR surface staining suggest that the maintenance of the synaptic surface expression of GluR2 requires various interactions among non-PDZ proteins, PDZ proteins, and GluR2. Further, those interactions appear to affect the GluR2 subunit selectively.

The amount of internalized GluR2 during 10 min did not differ significantly between the Tmub1/HOPS-RNAi and control neurons (Figures 5A, B), suggesting that Tmub1/HOPS is not related to the endocytosis of GluR2 in the steady state. Recycling of the internalized GluR2 to the cell surface was significantly delayed in the Tmub1/HOPS-RNAi neurons (Figures 5A, C), indicating that Tmub1/HOPS is related to the pathway by which GluR2 is recycled to the cell surface. Likewise, the inhibition of the NEEP21-GRIP [31] and GRIP-PICK1 [39] interactions delays the recycling of GluR2, suggesting that they are required for the recycling of GluR2 back to the plasma membrane. Consistent with our results of immunostaining of the surface endogenous GluR1, the internalization (Figures 5D, E) and recycling (Figures 5D, F) of GluR1 did not differ significantly between the Tmub1/HOPS-RNAi and control neurons. The inhibition of the NEEP21-GRIP interaction also did not affect the internalization and the recycling of GluR1 [31]. Therefore, Tmub1/HOPS appears to regulate the recycling of GluR2, which also requires multiple interactions such as those of NEEP21-GRIP and GRIP-PICK1.

The results from the neurons overexpressing Tmub1/HOPS were consistent with those from the Tmub1/HOPS-RNAi-transfected neurons. The internalization of GluR2 did not change (Figures 6A, B) but the recycling of GluR2 varied significantly (Figures 6A, C), supporting that Tmub1/HOPS is related to the recycling of GluR2 but not to the internalization of GluR2. The level of GluR2 or GluR1 recycled to the surface in the control cells (Figures 5C, 5F, 6C) appeared to be relatively low as compared to that in other reports [31], [39], although the time scales used by us were slightly different. In the absence of neuronal activity, internalized AMPARs are sorted for either degradation or reinsertion at synapses [24], [29]; however, upon incubation with AMPA or NMDA/TTX, AMPARs are more actively directed into the recycling pathway [29], [30]. Because we did not use any artificial stimulation in our recycling assay, some AMPARs may spontaneously be sorted for lysosomal degradation, which was represented as a rather lower recycling ratio in the control cells, as compared to other reports.

In surface-receptor regulation, Tmub1/HOPS appears to act contrary to ubiquitin and the UBL protein SUMO. While ubiquitin and SUMO decrease the surface number of their target receptors [42]–[44], Tmub1/HOPS increases the surface number of AMPARs. Like Tmub1/HOPS, some other UBL domain-containing proteins, such as Plic-1/ubiquilin-1 and GABARAP/ubiquilin-2, also increase the surface number of their receptors although the underlying mechanisms may be somewhat different. Tmub1/HOPS and GABARAP/ubiquilin-2 increase the surface expression of receptors by facilitating the trafficking of the receptors to the cell surface [11], while Plic-1/ubiquilin-1 increases the surface expression of receptors by increasing the stability of the receptors in the intracellular compartment [9]. Although the function of the UBL domain of Tmub1/HOPS remains to be revealed in future studies, it is interesting to speculate that the UBL domain can act as “pseudo” ubiquitin, which blocks ubiquitin function, similar to the dominant negative form of ubiquitin.

It was indicated that a large portion of Tmub1/HOPS exists at recycling endosomes rather than at early endosomes (Figure 7A, B). Internalized AMPARs for recycling enter to early endosomes and sorted to recycling endosomes for returning to the plasma membrane. NEEP21 interacts with the complex of GluR2 and GRIP at early endosomes and sorts the complex to the recycling pathway [31]. The recycling of GluR2-containing AMPARs appears to be carried out by the association with endosomal proteins and peripheral factors throughout the pathway. Tmub1/HOPS may act for the returning of GluR2-containing AMPARs to the plasma membrane, at recycling endosomes. Further, as the fluorescent intensity of intracellular GluR2 was not significantly changed (Figure 7C), the spatiotemporal information of AMPARs after the stimulation is suggested to be consistent with previous reports [29], [30].

**Figure 8:**
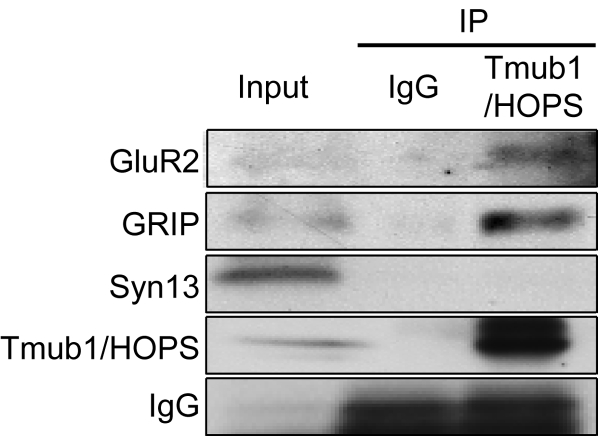
GluR2 and GRIP are immunoprecipitated from mouse brain extracts by Tmub1/HOPS. Immunoprecipitation (IP) from the mouse brain extract by using the anti-Tmub1/HOPS rabbit polyclonal antibody. GluR2 as well as GRIP, which has been known to interact with GluR2, were coimmunoprecipitated by Tmub1/HOPS. Syntaxin 13, which regulates transferrin receptor recycling, was not coimmunoprecipitated by Tmub1/HOPS.

Tmub1/HOPS only affected the GluR2 subunit and not the GluR1 subunit. Subunit-specific trafficking of AMPARs has been known to occur [23], [24] and appears to be critically related to their intracellular C-terminal-binding partners [20]. Interestingly, postsynaptic subunit-specific regulation of AMPARs is also affected by neurotransmitter release, i.e., a presynaptic effect [45]. Because Tmub1/HOPS is found at postsynaptic sites, Tmub1/HOPS is likely to be related to the postsynaptic complex containing GluR2 C-terminal-binding partners or GluR2, for the regulation of GluR2. GRIP, known to interact with the C-terminal site of GluR2 [32], was coimmunoprecipitated together with GluR2 by Tmub1/HOPS from the mouse brain lysate (Figure 8). GRIP is known to interact with other proteins, such as kinesin [14], PICK1 [39], NEEP21 [31]; further, it plays certain roles in the regulation of AMPAR recycling in order to modulate the level of synaptic receptors [23], [31], [46], [47]. The GluR2-GRIP complexes may associate with Tmub1/HOPS at some points of the constitutive recycling pathway. Tmub1/HOPS and GRIP were coimmunoprecipitated in HEK293 cells while they did not interact in our yeast 2-hybrid system (data not shown). For the association of Tmub1/HOPS with the complexes containing GluR2 and GRIP, it may be required another factor which exists in the HEK293 cells but not in the yeast. The relation among Tmub1/HOPS, GRIP, and GluR2 in the recycling of GluR2-containing AMPARs remains to be answered in future studies.

Although many questions, such as the molecular mechanisms by which Tmub1/HOPS works, are yet to be determined, our present results suggest that Tmub1/HOPS plays a role in helping GluR2 recycling to the cell surface. Because of its abundant and wide expression in the brain, Tmub1/HOPS might also participate in other functions in the brain. The trafficking of AMPARs is rapid and is required for the retention of synapse, which is related with the maintenance of memory. This study may eventually serve to the elucidation of the recycling pathway of AMPARs, which contributes to appropriate mental function.

## Materials and Methods

### *In silico* analysis

All the UBL domain-containing genes in the genomes of *Homo sapiens* and *Mus musculus* were obtained from the Celera database (database currently not available). The data obtained was confirmed with the UCSC genome browser database (http://genome.ucsc.edu) and NCBI Genbank (http://www.ncbi.nlm.nih.gov). These UBL domain-containing proteins were examined by the hmmpfam program that uses the Hidden Markov Model (HMM) database HMMER 2.2 g (http://hmmer.wustl.edu). These Pfam ubiquitin scores were higher than 1.0. From among the proteins that contained the UBL domains, we further selected UBLs using FANTOM3 databases and connected each representative transcript and protein set by public cDNA sequences, BLASTN and TBLASTN (http://www.ncbi.nlm.nih.gov). The results of hydropathy analysis from SOSUI (http://bp.nuap.nagoya-u.ac.jp/sosui/) were adapted to the proteins screened by the above method.

### Cloning and expression vectors

Mouse Tmub1/HOPS cDNAs were generated from the total RNA using Superscript II reverse transcriptase enzyme (Invitrogen, Carlsbad, CA), followed by PCR using the forward primer 5′-GTGCCATGGCCTTGATTGAA-3′ and the reverse primer 5′-GCGCCTTGGGGAATGA-3′. The PCR product was ligated into pCRII-TOPO (Invitrogen), and the full-length Tmub1/HOPS sequence was confirmed using an ABI PRISM 3700 (Applied Biosystems). Tmub1/HOPS was cloned using the Gateway™ system.

To create Tmub1/HOPS-RNAi expression vectors, the following oligonucleotides were annealed and ligated into pSUPER retro (Oligoengine, Seattle, WA) between the *Bgl*II and *Hind*III sites: 5′-gatccccGACACCATTGGCTCCTTAAttcaagagaTTAAGGAGCCAATGGTGTCttttta-3′ and 5′-agcttaaaaaGACACCATTGGCTCCTTAAtctcttgaaTTAAGGAGCCAATGGTGTCggg-3′ for Tmub1/HOPS-RNAi (519–537); 5′-gatccccGCCTGGGTCTCAACACATAttcaagagaTATGTGTTGAGACCCAGGCttttta-3′ and 5′-agcttaaaaaGCCTGGGTCTCAACACATAtctcttgaaTATGTGTTGAGACCCAGGCggg-3′ for Tmub1/HOPS-RNAi (134–152); 5′-gatccccGAAATCGGCAGCCTTCTGTttcaagagaACAGAAGGCTGCCGATTTCttttta-3′ and 5′-agcttaaaaaGAAATCGGCAGCCTTCTGTtctcttgaaACAGAAGGCTGCCGATTTCggg-3′ for Tmub1/HOPS-RNAi (732–750); 5′-gatccccTATAGACACTCTCGCGCGAttcaagagaTCGCGCGAGAGTGTCTATAttttta-3′ and 5′-agcttaaaaaTATAGACACTCTCGCGCGAtctcttgaaTCGCGCGAGAGTGTCTATAggg-3′ for the scramble controls. For EGFP or EGFP-Tmub1/HOPS overexpression in the hippocampal cultured neuron, we used *vesl-1* minimal promoter, which is neuron-selective (unpublished data) [48].

### Animals

All procedures related to the care and treatment of animals were in accordance with the guidelines of the National Institute of Health and the Animal Care and Use Committee (Mitsubishi Kagaku Institute of Life Sciences). C57BL/6J mice and Wistar SD rats were used in this study.

### Antibodies

For the production of the rabbit polyclonal antibody for Tmub1/HOPS, a fusion protein containing 29–191 aa of Tmub1/HOPS attached to a GST tag at the N terminus was purified on a GST column and used as an antigen. The antibody was purified by affinity chromatography using a HiTrap NHS-activated column (GE Healthcare, Uppsala, Sweden) coupled with maltose-binding protein-Tmub1/HOPS fusion proteins. The following antibodies were also used: anti-FLAG, anti-Actin, anti-MAP2, and anti-Alpha tubulin monoclonal antibodies (Sigma, St. Louis, MO, USA); anti-GRIP and anti-Rab4 monoclonal antibodies (BD Transduction Laboratories, Lexington, KY, USA); anti-GluR2, anti-Synaptophysin, and anti-Synaptotagmin monoclonal antibodies (Chemicon, Temecula, CA, USA); anti-Tau1 monoclonal antibody (MAB3420, Chemicon); anti-GluR1 rabbit polyclonal antibody (Calbiochem, La Jolla, CA, USA); anti-PSD-95 clone K28/43 mouse monoclonal antibody (Upstate Cell Signaling Solutions, Lake Placid, NY); and anti-Syntaxin 13 polyclonal antibody (Synaptic Systems, Göttingen, Germany).

### Northern blotting, Western blotting, and Subcellular fractionation

Total RNA from the mouse brain homogenates was isolated using Sepasol (Nacalai tesque, Kyoto, Japan), according to the manufacturer's instructions. Western blotting was performed as described previously [49]. For the isolation of the crude membrane fraction of mouse brain, brains from the C57BL/6J mice were homogenized in a buffer containing 250 mM sucrose, 3 mM imidazole, 1 mM EDTA and protease inhibitors and were centrifuged at 1,000×*g* for 10 min. The supernatant was further separated by ultracentrifugation at 100,000×*g* for 1 h. The resultant pellet and supernatant were used as the crude membrane and cytosol fractions, respectively. For subcellular fractionation of the mouse brain for synaptic protein, we performed following steps. Briefly, mouse brains were homogenized in a buffer (Buffer A) containing 4 mM HEPES (pH 7.4), 320 mM sucrose at 600 rpm for 10 times at 4°C and then centrifuged at 800×*g* for 10 min. The portion of the resultant pellet (P1) and supernatant (S1) were saved for western blotting. S1 was centrifuged at 9,200×*g* for 15 min. The resultant pellet (P2) and supernatant (S2) were saved. P2 diluted with Buffer A was centrifuged at 10,200×*g* for 15 min. The resultant pellet was further diluted with Buffer A and ice-chilled water was added to it. The diluted P2 was homogenized at 1,500 rpm 3 times. After chilling on ice for 30 min, the sample was centrifuged at 25,000×*g* for 20 min. The resultant supernatant (LS1) was further ultracentrifuged at 165,000×*g* for 2 h and the pellet was saved for the LP2 fraction. The resultant pellet (LP1) was centrifuged at 19,000×*g* for 150 min with a swing rotor. The obtained crude SPM fraction was further ultracentrifuged at 15,000×*g* for 30 min and then, the resultant pellet was saved for the SPM fraction. After addition of 0.5% TX-100, the sample was chilled on ice for 15 min and then centrifuged at 35,000×*g* for 20 min. The resultant supernatant and pellet were saved. The pellet was further treated with 1% TX-100 on ice for 15 min and then centrifuged 201,800×*g* for 1 h and the resultant supernatant and pellet were saved for western blotting.

### Electrophysiology

For recording of the mEPSC, the culture medium was exchanged for a saline solution containing 168 mM NaCl, 2.4 mM CaCl_2_, 1 mM MgCl_2_, 10 mM glucose, 10 mM HEPES, 0.5 µM TTX, 100 µM APV and 50 µM Bicuculline (pH 7.3), as reported previously [15], [19]. The patch electrode (4–6 MΩ) was filled with the whole-cell pipette solution containing 140 mM CsCl, 0.1 mM CaCl_2_, 5 mM MgCl_2_, 0.2 mM EGTA, 5 mM ATP, and 10 mM HEPES (pH 7.3). The whole-cell recording configuration from neurons expressing EGFP was achieved using an EPC-7 amplifier (HEKA, Germany) and a Digidata 1200 acquisition board (Axon Instruments). The membrane potential was clamped at −70 mV and +50 mV, and the signals were filtered at 10 kHz with a gain set of 0.5 mV/pA for 40 s recording periods. In all instances, the cells were excluded from the analysis if a leak current >200 pA was observed. The membrane resistance (Rm), series resistance (Rs), and membrane capacitance (Cm) were monitored. Only those recordings that had an Rm>125 MΩ and an Rs<15 MΩ were included in the analysis (the mean Rm, Rs, and Cm did not differ (two tailed *t*-test; *P*>0.05) among or within cells that were compared; CNQX (50 µM), an AMPAR antagonist, was bath-applied during a subset of recordings in order to determine that the detected mEPSC events were mediated by the AMPARs). The frequency, amplitude, rise time, and decay time of mEPSC were measured for a period of 40 s. mEPSCs were detected by setting the amplitude threshold to background as 3 times the background noise level (In all electrophysiological experiments, a similar amount of data was acquired from scramble and Tmub1/HOPS-RNAi neurons on the same day). All electrophysiological experiments were performed from at least 3 different platings of neurons from 2 different transfections. Rectification index (RI) was determined as the mean amplitude of the mEPSC at positive holding potential (+50 mV) divided by the mean amplitude of the mEPSC at negative holding potential (−70 mV).

### Cell culture and immunocytochemistry

Hippocampal neurons were prepared as described [19]. The cultured cells were transfected with 1 µg of DNA using Lipofectamine 2000 (Invitrogen, USA) on 13–14 DIV and were used on 15–16 DIV for immunostaining and electrophysiological recordings. Immunocytochemistry was performed as described [50] with some modifications. Briefly, cells were fixed with 4% paraformaldehyde/4% sucrose/phosphate-buffered saline (PBS) for 20 min at room temperature (RT) and then washed three times with PBS for 5 min. The cells were permeabilized with 0.1% TX-100/ PBS for 10 min at RT and blocked with blocking reagent (5% goat/1% BSA/0.1% NaN_3_/0.1% TX-100/PBS) for 30 min at RT and incubated with primary antibody diluted with blocking reagent for O/N at 4°C. After washing the primary antibody three times with PBS for 5 min, the cells were incubated with secondary antibody diluted with blocking reagent and then washed with PBS three times for 10 min.

For endogenous AMPAR staining, live hippocampal neurons were labeled for 10 min at 37°C with an antibody (10 µg/ml) directed against the extracellular region of either of the AMPAR subunits GluR1 and GluR2. After washing, the neurons were fixed for 8 min at RT and were washed with PBS. Then, the neurons were permeabilized for staining the presynaptic proteins VAMP2 and synaptophysin.

For the recycling assay, live neurons were surface labeled with mouse anti-GluR2 or anti-GluR1 antibodies, washed, and returned to the incubator for another 10 min to allow internalization. After the incubation, the antibodies remaining on the surface were stripped using an acid buffer (0.5 M NaCl/0.2 M acetic acid) on ice for 4 min [51]. The medium was then replaced with the culture medium and was returned to the incubator to allow resurfacing of the internalized receptor/antibody complex. Finally, the neurons were fixed and stained with Alexa 633-conjugated anti-mouse IgG antibody for 30 min at RT under impermeable conditions. Then, for the visualization of intracellular GluR2, the cells were permeabilized and stained with Alexa 568 conjugated anti-mouse IgG antibody for another 30 min under permeable conditions. All the recycling assays of GluR2 were performed at least three times, and the recycling assays of GluR1 were performed two times.

The immunostaining of internalized GluR2 and Tmub1/HOPS in Figure 7 was performed as described [31] with some modifications. Briefly, neurons were incubated for 1 h at 37°C with 2 µM TTX and for 10 min with TTX and anti-GluR2 antibody to label surface GluR2. Neurons were washed and then stimulated with 0 or 25 µM NMDA/TTX for 3 min, and then washed and further incubated for the indicated durations. Neurons were washed with PBS/30 mM glycine pH 2.5 to remove the remaining surface label, fixed, and immunolabeled using antibodies against Tmub1/HOPS or VAMP2.

### Image analysis

The images were captured on FluoView FV1000 (Olympus, Tokyo, Japan) or LSM510 version 3.2 (Carl Zeiss, Jena, Germany) confocal laser-scanning microscope and were analyzed using the FV1000 and LSM510 software. For picture presentations, seven optical sections acquired at 0.58 µm intervals were assembled as a Z-stack projection or a single section was used. For the image analysis, only a single section, which corresponded to a section at almost the same level relative to the upper and lower extremities, was used. For the quantitative analysis of synaptic AMPAR, presynaptic marker-positive puncta were defined as synaptic puncta and the fluorescence intensity of the puncta colocalized or attached to the synaptic puncta were measured. The fluorescence intensity was normalized by the AMPAR immunofluorescence intensity of EGFP-overexpressing neurons. For the quantitative analysis in the recycling assay, a 20-µm length of dendrites within an 80-µm radius from the center of the cell body was measured. The measured fluorescence intensity was normalized by the internalized AMPAR, in the scramble-transfected neurons or EGFP-overexpressing neurons during the first 10 min. For analyzing colocalization in Figure 7, colocalization was defined as the pixels that are positive for both GluR2 and Tmub1/HOPS or VAMP2. The weighted colocalization coefficient in the Y-axis of the graph corresponds to the value of the colocalized pixels, which reflect the intensity of the pixel, divided into the sum of the Tmub1/HOPS or VAMP2 pixels that reflect the intensity.

### Immunoprecipitation

For the immunoprecipitation assay of endogenous proteins, two whole mouse brains were dissected and homogenized in 10 volumes (of brain tissues) of a brain lysis buffer containing 20 mM Tris-HCl (pH 7.4), 100 mM NaCl, 1% Triton X-100, and protease inhibitors, using a glass-Teflon homogenizer at 3000 rpm×10 strokes at 4°C. Sonication was performed 6 times for 10 s each. Following ultracentrifugation at 100,000×g for 30 min, the supernatant was used for immunoprecipitation. The lysate was incubated with protein G-Sepharose beads for 1 h at 4°C to clarify nonspecific binding. Further, 10 µg of the anti-Tmub1/HOPS antibody or control rabbit IgG was added to 2 mg of the clarified supernatant. After incubation for 2 h at 4°C, 20 µl of protein G-Sepharose beads were added followed by further incubation for 1.5 h at 4°C. The beads were then spun down and washed 3 times with 6 volumes of the IP buffer. Immunoprecipitation experiments were performed at least 3 times.

## Supporting Information

Table S1 FANTOM3 expression profile of mouse UBLs. The expression of 57 UBLs, whose Pfam ubiquitin scores were higher than 1.0, was investigated by using the FANTOM3 database. Among them, 28 UBLs were revealed to have expression in the tissue containing neurons (bold). Tmub1/HOPS is written with bold italic characters. Abbreviations: adp, adipose; asN, activated spleen from NOD.Cz Idd3; cor, cortex; cqd, corpora quadrigemina; crb,cerebellum; edr, embryonic body below diaphragm region; eye, eyeball; fte, in vitro fertilized eggs; hed, head; hip, hippocampus; hrt, heart; htl, hypothalamus; kid, kidney; Lbm, LP S-treated bone marrow; liv, liver; lng, lung; mob, medulla oblongata; oau, ovary and uterus; pcr, pancreas; plc, placenta; prh, parthenogenote; sin, small intestine; skn, skin; spc, spinal cord; spg, sympathetic ganglion; spl, spleen; stm, stomach; tes, testis; thy, thymus; ton, tongue; vcr, visual cortex; wbd, whole body; wds, wolffian duct includes surrounding region.
(0.09 MB DOC)
