# Supplementary material for: Transmembrane and Ubiquitin-Like Domain-Containing Protein 1 (Tmub1/HOPS) Facilitates Surface Expression of GluR2-Containing AMPA Receptors
Source: PLoS One. 2008 Jul 30;3(7):e2809. doi: 10.1371/journal.pone.0002809 (PMC2474703; doi:10.1371/journal.pone.0002809)
Supplement: Table S1 — FANTOM3 expression profile of mouse UBLs. The expression of 57 UBLs, whose Pfam ubiquitin scores were higher than 1.0, was investigated by using the FANTOM3 database. Among them, 28 UBLs were revealed to have expression in the tissue containing neurons (bold). Tmub1/HOPS is written with bold italic characters. Abbreviations: adp, adipose; asN, activated spleen from NOD.Cz Idd3; cor, cortex; cqd, corpora quadrigemina; crb,cerebellum; edr, embryonic body below diaphragm region; eye, eyeball; fte, in vitro fertilized eggs; hed, head; hip, hippocampus; hrt, heart; htl, hypothalamus; kid, kidney; Lbm, LP S-treated bone marrow; liv, liver; lng, lung; mob, medulla oblongata; oau, ovary and uterus; pcr, pancreas; plc, placenta; prh, parthenogenote; sin, small intestine; skn, skin; spc, spinal cord; spg, sympathetic ganglion; spl, spleen; stm, stomach; tes, testis; thy, thymus; ton, tongue; vcr, visual cortex; wbd, whole body; wds, wolffian duct includes surrounding region. (0.09 MB DOC) [file pone.0002809.s001.doc]

| Name | Celera accession | FANTOM clone | expression pattern |
| --- | --- | --- | --- |
| ubiquitin A | mCG23116 | 0610008D13 | kid |
| ribosomal protein S27a | mCG13441 | 0610006J14 | kid |
| ubiquitin B | mCG23377 | 1700124N12 | **hed**, spl, tes, wbd |
| ubiquitin C | mCG16961 | 2510015A11 | liv, lng, kid, wbd |
| Rad23b | mCG20056 | G530128H04 | asN, edr, **hed**, hrt, Lbm, liv, lng |
| Rad23a | mCG5895 | 2810026G11 | kid, **mob**, prh, thy, wbd |
| UBL4 | mCG21230 | I530030L07 | **cer**, plc |
| BAT3 | mCG15922 | 7120447D04 | **spg**, wbd |
| ubiquilin-1(Plic-1) | mCG7066 | C330020N12 | asN, **hed**, kid |
| G1p2 | mCG22597 | 2900034J12 | **hip** |
| SF3A1 | mCG9630 | 4933425K18 | **cer**, lng, sin, skn, tes, thy |
| OASL-1 | mCG141547 | I830016G04 | asN, eye, Lbm, kid |
| ubiquilin-2 | mCG6755 | 3200002H06 | **hed** |
| ubiquilin-3 | mCG64525 | 4933400K24 | tes |
| Parkin | mCG7574 | 4930538K15 | tes |
| UHRF1 | mCG22967 | I920079B14 | hrt, Lbm, spl, stm |
| ANUBL1 | mCG129107 | E430036P15 | tes, wbd |
| Fau | mCG11741 | 4922505D07 | Lbm, kid, tes, thy |
| Sumo-1 | mCG116538 | 2510040N03 | liv, kid |
| unnamed | mCG21723 |  |  |
| ubiquilin-4 | mCG8833 | F830028B02 | asN, Lbm, wbd |
| UHRF2 | mCG6755 | B430210O11 | adp, asN, **crb**, **spc**, thy |
| Ubtd1 | mCG14288 | 6820406J02 | **mob** |
| HERPUD | mCG14659 | I420021O14 | **hed**, lng |
| UBPH | mCG3064 | C130061G12 | **hed**, wbd, wds |
| Sumo-2 | mCG146065 | 3426407D06 | **crb**, kid, plc, stm, tes, wbd |
| Sumo-3 | mCG3381 | G370085A19 | Lbm, **mob**, kid, sin, wbd |
| DC-UbP | mCG68025 | 4930571L24 | **cer**, tes |
| Fat10 | mCG23392 | 2010309G22 | sin |
| MGC20470 | mCG53928 | 4922504M18 | tes |
| ***Tmub1/HOPS*** | mCG21372 | 3110065C03 | **hed**, sin |
| UBL5 | mCG67928 | D130006N16 | kid, pcr, **spc**, ton, wbd |
| unnamed | mCG147873 | 1700011N24 | tes |
| elongin B | mCG55628 | 0610040H15 | Lbm, **hip**, kid, sin, wbd |
| SACS | mCG124983 | 3222404B11 | **cqd**, **hed** |
| BAG-1 | mCG9811 | F630037A18 | **hip**, ton |
| MGC10067 | mCG20094 | G270019L10 | **cor**, lng, liv, **htl** |
| FLJ22313 | mCG18376 | 5031400M07 | **cer**, fte, oau |
| midnolin | mCG13369 | F630044M20 | **hed**, **spc** |
| unnamed | mCG16505 | 2010004O20 | sin |
| BMSC-UbP | mCG9295 | F630040G07 | ton |
| unnamed | mCG68019 |  |  |
| UBL3 | mCG145043 | I530017L07 | **cer**, **mob**, plc, wbd |
| USP31 | mCG120445 | 6530419P07 | **cer**, **mob** |
| UBCE7IP3 | mCG13181 | I730030C10 | kid, lng, thy |
| IKK-beta | mCG6974 | G730008F11 | **cqd**, kid, lng, **spc**, wbd, |
| PLAC2 | mCG56863 |  |  |
| MGC3123 | mCG21372 | 3110065C03 | **hed**, sin |
| OASL2 | mCG141548 | 2310065A10 | Lbm, ton, wbd |
| USP14 | mCG22359 | 4932411O22 | **cqd**, tes, thy, wbd |
| unnamed | mCG15222 |  |  |
| unnamed | mCG1048340 |  |  |
| unnamed | mCG54232 |  |  |
| unnamed | mCG7881 |  |  |
| unnamed | mCG9114 |  |  |
| unnamed | mCG48649 |  |  |
| unnamed | mCG67952 |  |  |
| unnamed | mCG1031578 |  |  |

**Table S1. FANTOM3 expression profile of mouse UBLs**

The expression of 57 UBLs, whose Pfam ubiquitin scores were higher than 1.0, was investigated by using the FANTOM3 database. Among them, 28 UBLs were revealed to have expression in the tissue containing neurons (bold). Tmub1/HOPS is written with bold italic characters. Abbreviations: adp, adipose; asN, activated spleen from NOD.Cz Idd3; cor, cortex; cqd, corpora quadrigemina; crb,cerebellum; edr, embryonic body below diaphragm region; eye, eyeball; fte, in vitro fertilized eggs; hed, head; hip, hippocampus; hrt, heart; htl, hypothalamus; kid, kidney; Lbm, LP S-treated bone marrow; liv, liver; lng, lung; mob, medulla oblongata; oau, ovary and uterus; pcr, pancreas; plc, placenta; prh, parthenogenote; sin, small intestine; skn, skin; spc, spinal cord; spg, sympathetic ganglion; spl, spleen; stm, stomach; tes, testis; thy, thymus; ton, tongue; vcr, visual cortex; wbd, whole body; wds, wolffian duct includes surrounding region.
